# Trends in Belgian cause-specific mortality by migrant origin between the 1990s and the 2000s

**DOI:** 10.1186/s12889-019-6724-2

**Published:** 2019-04-16

**Authors:** Katrien Vanthomme, Hadewijch Vandenheede

**Affiliations:** Interface Demography, Department of Social Research, Faculty of Economic and Social Sciences & Solvay Business School, Vrije Universiteit Brussel, Pleinlaan 2, 1050 Brussels, Belgium

**Keywords:** Belgium, Immigrants, Mortality, Cancer mortality, Inequalities, Socioeconomic position, Gender

## Abstract

**Background:**

Belgium has a large migrant community that is increasingly ageing. As migrants may have faced environmental and social exposures before, during and after migration, they may have experienced an accelerated epidemiological transition. Studying mortality differentials between the migrant and native population may therefore allow for a better understanding of the aetiology of diseases. While many studies have assessed migrant mortality, few have looked into the role of gender or the trend over time. Therefore, this study aims to probe into mortality differences between the native and migrant population for all major causes of death (COD) during the 1990s and 2000s. We will discriminate between all major migrant groups and men and women as they have different migration histories.

**Methods:**

Individually linked data of the Belgian Census, the National Register and death certificates for the periods 1991–1997 and 2001–2008 were used. Migrant origin was based on both own and parents’ origin, hereby maximizing the population with migrant roots. We included native Belgians and migrants from the largest migrant groups aged 25 to 65 years. Both absolute and relative mortality differences by migrant origin were calculated for the most common COD.

**Results:**

We generally observed a migrant advantage for overall, cause-specific and cancer-specific mortality, with infection-related cancer mortality being the only exception. The effect was particularly strong for lifestyle-related COD, non-western migrants, and men. Over time, mortality declined among native Belgian men and women, yet remained stable for several migrant groups. This converging trend was largely due to smoking and reduced reproductive behaviour among migrants.

**Conclusions:**

The migrant mortality advantage stresses that there is room for improvement in the area of health in Belgium. Since the largest differences between native Belgians and migrants were observed for lifestyle-related diseases, and there is a tendency towards convergence of mortality over time, primary prevention tackling the most vulnerable groups remains crucial. Moreover, efforts should be made to ensure equal access to health care among the social and cultural strata.

## Background

### Rationale of the study

As in other Western European countries [1], migrants constitute an important portion of the Belgian population [2, 3]. Belgium is a country with a long and diverse history of migration [3–5]. A large share of migrants is from neighbouring countries such as the Netherlands and France. In general, migrants from Dutch descent belong to the highest socioeconomic strata, whereas migrants from French descent belong to the lowest socioeconomic strata. Another large share are labour migrants who immigrated in the post-War period, as well as their spouses who immigrated later. This group mainly consists of Italians, Spanish, Turkish and Moroccan immigrants who are more likely to be low-educated and in low socioeconomic positions (SEP). More recent migration constitutes immigrants from countries with former colonial ties (i.e. Democratic Republic of Congo) and political refugees. The migrant community in Belgium is thus rather diverse in terms of origin, reasons to migrate and socioeconomic profile. Nowadays the large migrant population is increasingly ageing, especially the first-generation labour migrants. This involves new challenges with regards to the organization of health care and the management of migrants’ health needs [6–8]. Hence, thoroughly documenting migrants’ mortality patterns relative to that of the native population is crucial. In addition, men and women had a different migration history [9, 10]: men generally tended to migrate for work purposes while women later followed for reasons of family reunification [4, 10, 11]. Therefore it is important to analyse gender differences in these migrant mortality patterns as well.

Health and mortality are the result of the interaction between environment, lifestyle and genetics [12]. Hence, migration can be seen as a kind of natural experiment: compared with the native population who faces the environmental and social exposures in their home country only, migrants experience different exposures during their life course [13]: before migration in their home country, during migration, and after migration in the host country [1, 7, 12]. In this way, migrants (especially from non-western countries) may have been subjected to an accelerated epidemiological transition [1, 14]. In western society infectious-disease mortality became less prevalent over time, while mortality due to chronic conditions (e.g. cancer) became predominant [1]. Initially migrants are likely to be protected against this typically western mortality pattern; yet this advantage will probably decrease over time with the adaptation to a western lifestyle [10, 13, 15]. Studying mortality differentials between migrants and the host population allows for a better understanding of the aetiology of diseases [1], and the relative importance of genetics, early-life and later-life exposures in this aetiology [16].

### Findings of previous studies

Both in Belgium and internationally, many studies that have assessed the relationship between migrant origin and health have shown a migrant mortality paradox [1–3, 5, 9, 11, 14, 17]. Despite their often poorer SEP, migrants (at least first-generation migrants) tend to have a mortality advantage compared with the native population. One explanation brought forward in literature is that this is the result of a data artefact [3, 5, 18], yet previous research proved this to be wrong [11, 19, 20]. The mortality advantage could also be explained by a selection effect [3, 5, 9, 11, 15, 18]: in order to embark on and survive the oft-difficult migration journey [1, 11, 21] the migrant population consists of a selection of healthy people, while unhealthy migrants are likely to return to their home country [3, 5, 11, 14, 21]. The cultural aspect is also important in explaining the mortality advantage: migrants are likely to maintain the healthy food habits and lifestyle of their home country while residing in the host country, at least shortly after migration [3, 5, 11, 15, 17, 21]. At the same time, the host country may involve better hygienic circumstances and a better organized and more efficient health care system than in the country of origin, especially for immigrants from non-western countries [17, 21]. However, time is an important factor as disease risks often convergence by duration of residence or over migration generation to reach host-country levels [1, 6, 14, 16, 22].

### Study aims

While many studies have assessed migrant mortality, few studies have looked into the role of gender or into the trend over time. Therefore, the purpose of this study is twofold: firstly, we want to assess whether there still is a migrant mortality advantage in Belgium in the 2000s. We will give an overview of mortality differences between native Belgians and all important migrant groups in Belgium, for all major causes of death (COD) during the period 2001–2008. We will analyse all major COD and all major cancer sites in order to provide clues on the different mechanisms at play [14]. We have a special interest in cancer-specific mortality because due to its multi-causality (both infectious- and lifestyle-related) and often unknown aetiology, we may provide hints on the origin of specific cancer types. We will study mortality separately for all major migrant groups as we expect different mortality patterns between migrants from non-western and western origins. Moreover, we will analyse gender differences in these migrant mortality patterns. We hypothesize that mortality patterns might be different for men and women as they traditionally had different motivations for migration [3–5, 11, 17]. As men immigrated for employment reasons and women for family reasons, we assume a health selection effect may be less likely in the case of women. Secondly, we want to probe into the evolution of overall, cause- and cancer-specific mortality by migrant origin and see whether mortality differences between the native and the migrant population have narrowed or widened over time between the 1990s and 2000s. We assume that over time, with longer periods spent in the host country, absolute and relative mortality differences may narrow as for instance the lifestyle of the host country may have been adopted.

## Methods

### Dataset

Data used in this paper consist of individually linked data of the Belgian Census with the National Register and death certificates. In a first stage, the Belgian censuses of 1991 and 2001 were linked with Register data for the periods 1991–1997 and 2001–2008 for the total de jure population living in Belgium at the moment of the censuses. The census contains demographic and socioeconomic information for all Belgian residents. The linkage with the National Register allowed us to include all emigration and mortality during the study periods. In a second stage, cause-specific mortality was added for al Belgian residents who died during the study period through individual linkage with the death certificates.

### Variables

This study includes all Belgian residents aged 25 to 65 years old. The lower age limit was chosen in order to have enough power in terms of migrant population and causes of death and the upper age limit was chosen because of the low number of 65+ migrants in the 1990s and because we want to look at mortality patterns over time. The definition of migrant origin was based on a stepwise approach, combining both own and parents’ origin, hereby maximizing the population with migrant roots. For individuals that could be linked to their parents, we used the nationality at birth of the father as specified in the censuses. If the father’s origin was unknown or Belgian, we took the nationality at birth of the mother. In both cases, the migrant origin of individuals with at least one of his/her parents having its roots outside Belgium was based on the nationality at birth of the parent. However, if the individual could not be linked to his/her parents, or if this information was unknown, the individual’s nationality at birth was used to define his/her migrant origin. If the individual’s nationality at birth was unknown, his/her current nationality as available in census was used as a proxy for migrant origin. For this paper we included the largest migrant groups in Belgium (see also Table 1), i.e. migrants originating from the neighbouring countries (the Netherlands and France), Spain, Italy, Eastern Europe (Poland, Hungary, Romania, Czechoslovakia, Bulgaria, Czech Republic and Slovakia), Turkey, Morocco and Sub-Saharan Africa (SSA) (Congo (Zaire), Burundi and Rwanda). Since we will compare migrant mortality in the 1990s with the 2000s, we were not able to stratify our analyses by migrant generation as the number of second-generation migrants was too small in the 1990s. We will study mortality differences by migrant origin for the most common causes of (cancer) death, which were classified according to the International Statistical Classification of Diseases and Related Health Problems, the ninth revision for the 1990s and tenth revision for the 2000s (Table 2).

**Table 1:** Number of persons by migrant origin and gender

	Men		Women	
	1990s	2000s	1990s	2000s
Belgium	2,314,735	2,280,006	2,323,508	2,273,845
Netherlands	28,993	46,459	28,559	42,556
France	41,840	59,278	46,771	61,990
Spain	19,334	11,861	19,437	10,446
Italy	91,138	109,009	77,861	95,323
Eastern Europe	14,600	29,084	15,269	34,098
Turkey	18,977	33,575	16,145	29,959
Morocco	34,238	62,756	26,106	51,301
Sub-Saharan Africa	5454	20,075	5067	20,795

**Table 2:** Causes of death, corresponding ICD-codes and total number of deaths

	1990s			2000s		
	ICD-9	Men	Women	ICD-10	Men	Women
All Deaths	001–999	113,148	59,127	A00-Y98	96,926	52,878
Cancer	140–239	41,374	26,806	C00-C99	34,534	23,941
Head and neck	140–149; 160–161	3223	578	C00–14; C30–32	2615	504
Oesophagus	150	1473		C15	1633	
Stomach	151	1539	655	C16	1126	481
Colorectal	153–154	3398	2507	C18–21	2855	1948
Liver	155	880		C22	1029	
Pancreas	157	1691	1037	C25	1715	1119
Lung	162	16,460	2919	C33–34	12,561	4100
Breast	174–175		8010	C50		6604
Uterus	179; 182		757	C54–55		610
Ovary	183		2104	C56		1587
Prostate	185	1637		C61	1138	
Bladder	188	1185		C67	869	
Brain	190–192	1613	1164	C69–72	1250	873
Leukaemia	204–208		737	C91–95		555
Circulatory	390–459	29,352	12,354	I00-I99	21,887	9397
Respiratory	460–519	7599	2789	J00-J99	5853	2938
Digestive	520–579	5523	3006	K00-K93	5837	2889
Injuries	800–999	14,469	5729	S00-T99	8200	3259

### Statistical analyses

In this paper we will calculate both absolute and relative measures of migrant mortality inequalities. We calculated for each migrant origin group the cause-specific mortality rates in the 1990s and the 2000s by gender. To account for differences in the age structure over time and between migrant groups, the cause-specific mortality rates by gender and migrant group in 1991–1997 and 2001–2008 were directly standardized to the total Belgian population aged 25 to 65 years in 2001. We then compared the age-standardized mortality rates (ASMR) and the 95% confidence intervals of the different migrant origins with the ASMR of the native Belgians to assess whether there were mortality differences. To assess the evolution in absolute mortality patterns by migrant origin, the percentage of change in mortality in the 2000s towards the 1990s were calculated for all COD. The significance of the trend over time was formally tested as explained by Altman & Bland [23]. Furthermore, for both periods, relative mortality inequalities were calculated for each migrant group compared with the native Belgians. These mortality rate ratios (MRR) are the result of Poisson models adjusted for attained age. To test the gender hypothesis, all analyses were stratified by gender. All analyses have been performed using Stata/MP 14.2.

## Results

### Differences in overall mortality and large causes of death by migrant origin

When we look at the overall and cause-specific ASMR (Tables 3 and 4), we generally observed a mortality advantage among the migrant groups. However, French migrants as well as Eastern European male migrants had higher overall mortality compared with the native Belgian population. For instance, in the 2000s in relative terms (Tables 5 and 6), Eastern European men had a mortality excess of 7% (MRR: 1.07; 95% CI 1.02–1.13), while men and women from French descent had an excess of respectively 23% (MRR: 1.23; 95% CI 1.19–1.27) and 16% (MRR: 1.16; 95% CI 1.11–1.22). This excess mortality among migrants from French descent was mainly caused by their higher mortality from digestive diseases (in men and women), as well as respiratory diseases and cancer among French men.

**Table 3:** Age-standardized mortality rate per 100,000 person-years in the 2000s and the percentage change towards the 1990s, by migrant origin and cause of death - Belgian men aged 25–65 years

MEN	ASMR (95% C.I.)	% change	ASMR (95% C.I.)	% change	ASMR (95% C.I.)	% change	ASMR (95% C.I.)	% change	ASMR (95% C.I.)	% change
	Belgium		Netherlands		France		Spain		Italy	
All Deaths	420.4 (417.6–423.2)	**− 17.6**	315.2 (297.2–333.2)	**− 24.7**	519.3 (496.4–542.3)	**−19.8**	295.9 (256.0–335.8)	**−27.2**	341.9 (329.3–354.5)	**− 27.9**
Cancer	151.8 (150.1–153.5)	**−20.4**	124.4 (113.1–135.8)	**−17.5**	180.5 (166.7–194.3)	**− 23.3**	119.8 (94.0–145.7)	−11.1	123.8 (116.1–131.5)	**− 23.9**
Head and neck	11.8 (11.3–12.3)	**−23.9**	5.4 (3.0–7.8)	−40.7	19.7 (15.2–24.2)	**−44.8**	5.5 (1.0–9.9)	−54.5	5.0 (3.5–6.5)	−15.3
Oesophagus	7.5 (7.1–7.8)	7.1	6.4 (3.8–9.0)	−31.2	8.1 (5.2–10.9)	−36.7	2.1 (0.0–5.0)	0.0	2.5 (1.4–3.6)	−30.6
Stomach	4.8 (4.5–5.1)	**−31.4**	3.7 (1.7–5.6)	42.3	4.7 (2.5–6.9)	−6.0	6.6 (0.5–12.6)	−9.6	5.6 (4.0–7.2)	**−37.8**
Colorectal	12.6 (12.2–13.1)	**−20.3**	13.7 (9.9–17.4)	2.2	12.6 (9.1–16.2)	−29.6	9.8 (2.2–17.5)	36.1	10.5 (8.2–12.8)	0.0
Liver	4.2 (3.9–4.5)	**13.5**	2.9 (1.2–4.7)	−23.7	9.7 (6.7–13)	47.0	7.6 (1.2–14.0)	15.2	6.1 (4.4–7.8)	**−35.8**
Pancreas	7.4 (7.0–7.8)	−5.1	7.6 (4.8–10.4)	153.3	8.3 (5.4–11.2)	1.2	9.3 (2.5–16.1)	111.4	6.5 (4.7–8.3)	0.0
Lung	55.1 (54.0–56.1)	**−27.4**	41.9 (35.3–48.5)	**− 28.3**	66.4 (57.9–74.8)	**−22.3**	31.7 (17.9–45.5)	**−41.6**	49.7 (44.8–54.6)	**− 27.7**
Prostate	5.0 (4.7–5.4)	**−32.4**	5.6 (3.2–8.0)	60.0	5.4 (2.8–8.1)	−38.6	8.0 (1.0–14.9)	110.5	2.9 (1.7–4.1)	−31.0
Bladder	3.9 (3.6–4.2)	**−27.8**	3.7 (1.8–5.7)	−2.6	5.2 (2.7–7.6)	− 31.6	0.0 (0.0–0.0)	−100.0	3.3 (2.1–4.6)	−15.4
Brain	5.5 (5.2–5.8)	**−26.7**	5.0 (2.8–7.3)	−31.5	4.4 (2.4–6.4)	−34.3	5.1 (0.0–11.2)	− 30.1	4.5 (3.0–6.0)	−26.2
Circulatory	95.9 (94.6–97.3)	**−28.5**	69.0 (60.5–77.4)	**−41.3**	98.0 (87.8–108.2)	**− 28.3**	47.1 (30.7–63.4)	**− 35.2**	79.4 (73.2–85.5)	**−30.8**
Respiratory	25.9 (25.2–26.6)	**−21.3**	16.5 (12.4–20.7)	−13.6	33.1 (27.1–39.1)	**−25.8**	9.7 (3.3–16.0)	**−70.2**	20.8 (17.6–24.1)	**−62.5**
Digestive	25.9 (25.2–26.6)	0.4	11.9 (8.4–15.4)	25.3	34.5 (28.6–40.5)	−20.5	16.8 (7.5–26.1)	−8.7	15.3 (12.6–18.0)	−27.8
Injuries	34.6 (35.6–37.2)	**−45.8**	31.0 (25.4–36.6)	**−37.8**	24.2 (19.6–28.8)	**−67.6**	22.3 (11.8–32.7)	**−53.5**	7.6 (5.8–9.4)	**− 83.3**
	Eastern Europe		Turkey		Morocco		Sub-Saharan Africa			
All Deaths	446.3 (417.5–475.1)	**−18.8**	375.6 (344.0–407.2)	− 11.6	288.6 (270.9–306.3)	**− 16.9**	470.6 (410.3–530.9)	−24.7		
Cancer	148.0 (131.1–164.9)	−9.2	108.4 (90.9–125.9)	−12.9	94.8 (84.3–105.3)	− 2.0	131.0 (97.7–164.3)	70.1		
Head and neck	9.7 (5.4–14)	− 29.2	1.0 (0.0–2.4)	−86.5	2.2 (0.6–3.9)	−37.1	12.5 (2.2–22.8)	−0.8		
Oesophagus	4.1 (1.2–6.9)	0.0	0.7 (0.0–2.0)	−78.8	2.0 (0.4–3.6)	66.7	4.6 (0.0–9.3)	–		
Stomach	3.5 (1.1–6.0)	−30.0	5.8 (1.7–9.9)	−37.0	6.5 (3.8–9.2)	75.7	4.9 (0.0–11.6)	–		
Colorectal	10.6 (6.1–15.1)	1.9	3.1 (0.2–6.1)	−27.9	5.0 (2.6–7.4)	− 36.7	13.8 (1.7–26.0)	–		
Liver	3.4 (0.9–5.9)	78.9	5.2 (1.2–9.2)	23.8	3.8 (1.7–5.9)	−34.5	15.6 (5.4–25.9)	−47.5		
Pancreas	8.1 (4.3–12.0)	−2.4	6.5 (2.3–10.7)	−1.5	5.7 (3.1–8.3)	−3.4	11.4 (1.0–21.8)	–		
Lung	66.5 (55.1–78.0)	12.5	45.6 (34.1–57.1)	−6.2	40.6 (33.6–47.6)	25.3	27.6 (11.3–43.8)	93.0		
Prostate	3.0 (0.6–5.5)	−23.1	3.5 (0.0–7.0)	−35.2	5.5 (3.0–8.0)	27.9	13.1 (3.6–22.6)	4.0		
Bladder	3.3 (0.6–5.9)	−45.0	0.0 (0.0–0.0)	−100.0	1.4 (0.0–2.7)	−67.4	0.0 (0.0–0.0)	–		
Brain	3.6 (1.0–6.1)	−18.2	4.1 (1.1–7.1)	−31.7	3.0 (1.2–4.8)	−42.3	0.4 (0.0–1.1)	–		
Circulatory	95.9 (82.4–109.5)	**−36.0**	88.2 (72.7–103.7)	−13.7	61.9 (53.5–70.2)	−16.0	114.4 (83.0–145.8)	5.3		
Respiratory	22.8 (16.2–29.4)	**−50.0**	20.8 (13.3–28.3)	−1.4	13.5 (9.6–17.4)	4.7	29.1 (12.9–45.3)	**−72.1**		
Digestive	26.9 (19.8–34.0)	−24.0	6.5 (2.5–10.6)	− 27.0	6.2 (3.5–8.9)	0.0	12.8 (5.1–20.6)	−52.1		
Injuries	27.2 (20.5–33.9)	**−58.9**	23.1 (16.8–29.3)	**−40.6**	21.3 (17.2–25.4)	−22.8	17.1 (9.3–25.0)	**−74.6**		

Results in bold are significant at the 0.05-level

**Table 4:** Age-standardized mortality rate per 100,000 person-years in the 2000s and the percentage change towards the 1990s, by migrant origin and cause of death - Belgian women aged 25–65 years

Women	ASMR (95% C.I.)	% change	ASMR (95% C.I.)	% change	ASMR (95% C.I.)	% change	ASMR (95% C.I.)	% change	ASMR (95% C.I.)	% change
	Belgium		Netherlands		France		Spain		Italy	
All Deaths	221.1 (219.1–223.1)	**−12.5**	197.5 (182.8–212.2)	**−15.2**	255.4 (240.6–270.2)	−5.3	162.0 (129.8–194.3)	− 1.3	179.3 (169.7–189.0)	**−13.6**
Cancer	101.0 (99.7–102.4)	**−14.0**	91.1 (81.1–101.1)	**−21.0**	107.6 (97.9–117.2)	−0.7	72.1 (51.5–92.7)	17.6	81.0 (74.5–87.5)	−9.4
Head and neck	2.1 (1.9–2.3)	**−19.2**	1.9 (0.5–3.3)	−44.1	3.9 (2.0–5.7)	−7.1	3.7 (0.0–7.9)	362.5	1.2 (0.4–2.0)	9.1
Stomach	1.8 (1.7–2.0)	**−33.3**	1.4 (0.2–2.6)	−57.6	1.4 (0.3–2.4)	27.3	0.0 (0.0–0.0)	−100.0	3.5 (2.2–4.9)	9.4
Colorectal	8.2 (7.8–8.6)	**−23.4**	7.8 (4.8–10.7)	−39.1	8.5 (5.8–11.3)	−26.7	11 (1.5–20.4)	96.4	7.1 (5.2–9.0)	−29.7
Pancreas	4.6 (4.3–4.9)	4.5	3.5 (1.6–5.4)	−34.0	7.2 (4.7–9.7)	75.6	2.7 (0.0–6.5)	−15.6	5.1 (3.5–6.8)	41.7
Lung	17.4 (16.8–17.9)	**38.1**	20.2 (15.3–25.0)	4.7	20.7 (16.4–24.9)	**59.2**	15.6 (5.9–25.3)	79.3	12.1 (9.6–14.7)	**42.4**
Breast	28.2 (27.5–28.9)	**−22.7**	23.2 (18.1–28.3)	−12.1	28.6 (23.6–33.6)	−3.4	16.9 (7.2–26.6)	9.7	18.9 (15.8–22.0)	**−23.5**
Uterus	2.5 (2.3–2.7)	**−21.9**	1.6 (0.3–2.9)	−55.6	3.3 (1.6–5.0)	−8.3	3.7 (0.0–8.0)	27.6	2.4 (1.2–3.5)	−27.3
Ovary	6.8 (6.4–7.1)	**−27.7**	7.1 (4.3–9.8)	−15.5	6.7 (4.2–9.2)	−6.9	4.8 (0.0–9.6)	118.2	5.1 (3.5–6.8)	13.3
Brain	3.7 (3.4–4.0)	**−26.0**	2.1 (0.5–3.6)	−44.7	2.5 (1.1–4.0)	−41.9	2.8 (0.0–6.6)	−20.0	3.4 (2.1–4.7)	−40.4
Leukaemia	2.3 (2.1–2.5)	**−28.1**	3.6 (1.6–5.5)	28.6	3.5 (1.8–5.3)	29.6	1.5 (0.0–4.5)	−16.7	2.0 (1.0–3.0)	− 13.0
Circulatory	39.2 (38.3–40.0)	**−23.1**	36.2 (29.9–42.5)	−0.8	41.1 (35.1–47.1)	**−31.4**	19.0 (8.5–29.5)	−44.8	35.4 (31.1–39.7)	**−27.3**
Respiratory	12.5 (12.1–13.0)	**6.8**	11.6 (8.1–15.1)	−14.7	10.6 (7.6–13.6)	5.0	11.8 (2.2–21.3)	100.0	8.0 (6.0–10.1)	−7.0
Digestive	12.4 (11.9–12.9)	**−6.1**	7.6 (4.7–10.5)	15.2	19.8 (15.6–24)	−12.8	5.2 (0.0–11.6)	−30.7	8.5 (6.4–10.6)	−9.6
Injuries	14.1 (13.6–14.7)	**−44.3**	13.4 (9.6–17.63)	−33.3	8.8 (6.1–11.5)	**−66.3**	12.0 (4.1–19.9)	−13.7	2.4 (1.3–3.4)	**−87.4**
	Eastern Europe		Turkey		Morocco		Sub-Saharan Africa			
All Deaths	223.1 (203.8–242.5)	**−21.9**	218.2 (193.8–242.7)	3.1	184.3 (168–200.7)	**−19.5**	270.0 (230.9–309.0)	**−39.3**		
Cancer	100.9 (87.9–114.0)	**−22.7**	67.8 (54.4–81.1)	34.0	61.9 (52.6–71.2)	27.1	103.6 (76.7–130.5)	3.0		
Head and neck	3.2 (0.8–5.5)	−47.5	1.3 (0.0–3.2)	18.2	0.8 (0.0–1.7)	−38.5	0.7 (0.0–2.0)	−86.3		
Stomach	5.6 (2.5–8.6)	51.4	3.7 (0.6–6.7)	−33.9	4.7 (2.1–7.3)	9.3	3.4 (0.0–9.0)	100.0		
Colorectal	8.4 (4.6–12.1)	16.7	2.5 (0.0–5.0)	−28.6	6.4 (3.3–9.6)	68.4	16.6 (5.0–28.1)	876.5		
Pancreas	6.1 (2.8–9.4)	41.9	6.8 (2.3–11.3)	–	5.3 (2.4–8.2)	657.1	8.8 (0.0–18.5)	–		
Lung	20.0 (14.2–25.8)	69.5	8.3 (3.6–13.1)	23.9	4.3 (1.8–6.8)	377.8	10.6 (2.7–18.6)	79.7		
Breast	20.4 (14.6–26.3)	**−41.4**	10.5 (5.6–15.5)	208.8	14.2 (10.2–18.2)	40.6	24.4 (11.7–37.1)	−16.2		
Uterus	1.8 (0.2–3.4)	−5.3	5.7 (1.5–10.0)	–	1.1 (0.0–2.3)	22.2	0.4 (0.0–1.2)	−96.5		
Ovary	5.7 (2.6–8.8)	−47.2	4.7 (1.2–8.2)	−42.0	3.0 (1.0–5.0)	−31.8	5.6 (0.0–12.4)	− 50.4		
Brain	4.2 (1.6–6.9)	−53.8	4.4 (1.2–7.7)	−20.0	3.2 (1.0–5.5)	88.2	0.9 (0.0–2.1)	−92.4		
Leukaemia	1.2 (0.0–2.5)	−78.2	2.7 (0.0–5.6)	–	0.9 (0.0–1.9)	−62.5	8.7 (1.0–16.5)	–		
Circulatory	38.3 (30.1–46.5)	**−35.8**	52.4 (40.2–64.7)	19.1	42.7 (34.5–50.8)	**92.3**	45.9 (30.2–61.7)	−46.6		
Respiratory	7.3 (3.7–10.9)	−41.1	9.9 (4.5–15.3)	−37.3	9.8 (5.9–13.7)	−21.0	11.9 (3.5–20.3)	88.9		
Digestive	11.2 (6.9–15.5)	−30.4	4.7 (1.1–8.3)	−19.0	3.1 (1.0–5.2)	**−74.2**	11.8 (3.6–20.0)	87.3		
Injuries	12.1 (7.8–16.5)	**−50.0**	9.8 (5.5–14.2)	−24.6	10.0 (6.8–13.1)	− 5.7	11.5 (5.4–17.6)	−63.7		

Results in bold are significant at the 0.05-level

**Table 5:** Mortality rate ratio by migrant origin, cause of death and study period - Men living in Belgium aged 25–65 years

	Belgium	Netherlands	France	Spain	Italy	Eastern Europe	Turkey	Morocco	Sub-Saharan Africa
Men – 1990s									
All Deaths	ref.	0.82 (0.78,0.87)	1.28 (1.23,1.33)	0.84 (0.79,0.90)	0.90 (0.88,0.93)	1.09 (1.03,1.16)	0.75 (0.70,0.81)	0.65 (0.62,0.69)	1.05 (0.91,1.22)
Cancer	ref.	0.79 (0.73,0.86)	1.20 (1.13,1.28)	0.74 (0.66,0.83)	0.86 (0.81,0.90)	0.89 (0.80,0.99)	0.57 (0.50,0.66)	0.51 (0.46,0.56)	0.45 (0.30,0.67)
Head and neck	ref.	0.54 (0.37,0.77)	2.17 (1.86,2.55)	0.63 (0.41,0.96)	0.38 (0.29,0.48)	0.97 (0.67,1.39)	0.38 (0.21,0.69)	0.27 (0.17,0.44)	0.53 (0.17,1.66)
Oesophagus	ref.	1.34 (0.95,1.88)	1.56 (1.18,2.06)	0.39 (0.17,0.86)	0.47 (0.33,0.65)	0.57 (0.28,1.14)	0.47 (0.21,1.05)	0.22 (0.10,0.48)	–
Stomach	ref.	0.30 (0.14,0.63)	0.70 (0.46,1.08)	1.17 (0.74,1.87)	1.28 (1.04,1.59)	0.72 (0.39,1.35)	1.34 (0.82,2.19)	0.74 (0.47,1.16)	–
Colorectal	ref.	0.91 (0.68,1.21)	1.05 (0.83,1.33)	0.49 (0.30,0.79)	0.69 (0.57,0.83)	0.67 (0.44,1.03)	0.30 (0.15,0.60)	0.46 (0.31,0.67)	–
Liver	ref.	1.10 (0.65,1.87)	1.72 (1.17,2.51)	1.80 (1.08,3.00)	2.50 (2.02,3.08)	0.66 (0.28,1.60)	0.94 (0.42,2.11)	1.82 (1.22,2.70)	5.38 (2.22,13.00)
Pancreas	ref.	0.38 (0.21,0.71)	1.06 (0.76,1.49)	0.59 (0.32,1.09)	0.88 (0.69,1.12)	1.04 (0.64,1.70)	1.07 (0.63,1.81)	0.70 (0.45,1.09)	–
Lung	ref.	0.76 (0.66,0.88)	1.13 (1.02,1.26)	0.78 (0.65,0.92)	0.91 (0.84,0.98)	0.84 (0.70,1.00)	0.55 (0.43,0.70)	0.43 (0.36,0.51)	0.28 (0.12,0.67)
Prostate	ref.	0.55 (0.32,0.95)	1.11 (0.77,1.59)	0.56 (0.29,1.07)	0.53 (0.38,0.73)	0.54 (0.27,1.08)	0.28 (0.09,0.86)	0.51 (0.29,0.90)	1.77 (0.44,7.08)
Bladder	ref.	0.70 (0.39,1.23)	1.50 (1.05,2.15)	0.86 (0.46,1.61)	0.72 (0.52,1.01)	1.23 (0.71,2.12)	0.61 (0.25,1.46)	0.72 (0.42,1.25)	–
Brain	ref.	0.88 (0.58,1.32)	0.82 (0.57,1.18)	0.95 (0.58,1.55)	0.88 (0.69,1.11)	0.83 (0.47,1.46)	0.86 (0.50,1.49)	0.55 (0.34,0.88)	–
Circulatory	ref.	0.86 (0.78,0.95)	1.04 (0.95,1.12)	0.58 (0.49,0.67)	0.85 (0.80,0.90)	1.16 (1.04,1.30)	0.69 (0.59,0.81)	0.51 (0.45,0.58)	0.76 (0.52,1.10)
Respiratory	ref.	0.56 (0.43,0.72)	1.35 (1.16,1.57)	1.12 (0.90,1.39)	1.53 (1.40,1.68)	1.29 (1.05,1.60)	0.51 (0.35,0.75)	0.39 (0.29,0.53)	1.14 (0.57,2.27)
Digestive	ref.	0.42 (0.31,0.58)	1.65 (1.44,1.90)	0.75 (0.55,1.01)	0.78 (0.68,0.89)	1.42 (1.13,1.80)	0.27 (0.16,0.46)	0.22 (0.15,0.33)	0.47 (0.20,1.13)
Injuries	ref.	0.81 (0.70,0.92)	1.17 (1.06,1.28)	0.72 (0.60,0.86)	0.71 (0.66,0.77)	0.99 (0.84,1.18)	0.66 (0.56,0.79)	0.49 (0.42,0.57)	0.75 (0.54,1.03)
Men – 2000s									
All Deaths	ref.	0.76 (0.73,0.80)	1.23 (1.19,1.27)	0.71 (0.64,0.78)	0.78 (0.75,0.80)	1.07 (1.02,1.13)	0.74 (0.69,0.78)	0.68 (0.65,0.71)	0.87 (0.80,0.94)
Cancer	ref.	0.83 (0.77,0.89)	1.14 (1.07,1.21)	0.76 (0.64,0.89)	0.78 (0.74,0.82)	0.97 (0.88,1.07)	0.64 (0.56,0.72)	0.61 (0.56,0.66)	0.71 (0.61,0.84)
Head and neck	ref.	0.48 (0.34,0.68)	1.67 (1.40,1.99)	0.59 (0.31,1.13)	0.41 (0.32,0.52)	0.69 (0.47,1.01)	0.08 (0.03,0.25)	0.15 (0.08,0.27)	0.75 (0.45,1.25)
Oesophagus	ref.	0.89 (0.64,1.23)	1.14 (0.87,1.50)	0.32 (0.10,1.01)	0.30 (0.21,0.43)	0.47 (0.26,0.85)	0.05 (0.01,0.34)	0.23 (0.12,0.43)	0.88 (0.47,1.65)
Stomach	ref.	0.62 (0.38,1.02)	1.02 (0.70,1.47)	1.23 (0.59,2.59)	1.15 (0.90,1.46)	0.98 (0.58,1.65)	1.02 (0.59,1.76)	1.33 (0.95,1.86)	1.02 (0.48,2.14)
Colorectal	ref.	1.15 (0.92,1.44)	1.08 (0.86,1.34)	0.73 (0.41,1.33)	0.77 (0.64,0.92)	0.86 (0.61,1.22)	0.26 (0.13,0.51)	0.44 (0.31,0.63)	0.48 (0.24,0.97)
Liver	ref.	0.78 (0.49,1.24)	2.21 (1.69,2.88)	1.76 (0.91,3.40)	1.51 (1.21,1.89)	0.79 (0.42,1.46)	0.83 (0.43,1.60)	0.97 (0.63,1.48)	4.16 (2.78,6.23)
Pancreas	ref.	0.95 (0.69,1.30)	1.22 (0.93,1.59)	1.10 (0.59,2.05)	0.74 (0.58,0.93)	1.10 (0.74,1.64)	0.92 (0.58,1.46)	0.79 (0.55,1.11)	1.15 (0.65,2.03)
Lung	ref.	0.74 (0.64,0.84)	1.10 (0.99,1.23)	0.56 (0.41,0.78)	0.89 (0.82,0.96)	1.24 (1.08,1.42)	0.78 (0.64,0.94)	0.73 (0.64,0.84)	0.32 (0.21,0.49)
Prostate	ref.	1.35 (0.96,1.90)	0.80 (0.52,1.22)	1.30 (0.62,2.73)	0.68 (0.49,0.94)	0.68 (0.36,1.32)	0.52 (0.21,1.25)	1.24 (0.84,1.81)	3.22 (1.94,5.37)
Bladder	ref.	1.02 (0.66,1.57)	1.04 (0.69,1.57)	–	0.80 (0.58,1.11)	0.99 (0.55,1.80)	–	0.26 (0.11,0.63)	–
Brain	ref.	1.01 (0.71,1.44)	0.89 (0.63,1.26)	0.57 (0.21,1.53)	0.77 (0.59,0.99)	0.64 (0.36,1.13)	0.56 (0.31,1.02)	0.50 (0.32,0.79)	0.19 (0.05,0.74)
Circulatory	ref.	0.74 (0.67,0.82)	1.01 (0.93,1.10)	0.48 (0.37,0.63)	0.80 (0.75,0.85)	1.03 (0.92,1.16)	0.81 (0.71,0.93)	0.62 (0.55,0.69)	0.87 (0.73,1.04)
Respiratory	ref.	0.66 (0.54,0.81)	1.33 (1.16,1.54)	0.50 (0.30,0.84)	0.69 (0.60,0.79)	1.00 (0.79,1.26)	0.69 (0.51,0.93)	0.61 (0.49,0.76)	0.78 (0.53,1.16)
Digestive	ref.	0.45 (0.36,0.57)	1.28 (1.12,1.46)	0.60 (0.39,0.93)	0.53 (0.46,0.61)	1.01 (0.82,1.25)	0.21 (0.13,0.33)	0.20 (0.14,0.28)	0.53 (0.36,0.79)
Injuries	ref.	0.84 (0.73,0.97)	0.67 (0.58,0.77)	0.67 (0.48,0.94)	0.20 (0.17,0.24)	0.81 (0.68,0.97)	0.63 (0.53,0.75)	0.65 (0.57,0.74)	0.52 (0.40,0.68)

All models are adjusted for attained age

**Table 6:** Mortality rate ratio by migrant origin, cause of death and study period - Women living in Belgium aged 25–65 years

	Belgium	Netherlands	France	Spain	Italy	Eastern Europe	Turkey	Morocco	Sub-Saharan Africa
Women – 1990s									
All Deaths	ref.	0.92 (0.86,0.98)	1.08 (1.03,1.14)	0.67 (0.61,0.74)	0.78 (0.75,0.82)	1.16 (1.07,1.26)	0.75 (0.67,0.84)	0.77 (0.71,0.84)	2.18 (1.90,2.51)
Cancer	ref.	0.96 (0.87,1.06)	0.92 (0.85,0.99)	0.52 (0.44,0.62)	0.73 (0.68,0.78)	1.15 (1.02,1.29)	0.43 (0.35,0.54)	0.46 (0.39,0.55)	0.97 (0.71,1.33)
Head and neck	ref.	1.42 (0.84,2.41)	1.47 (0.97,2.22)	0.33 (0.08,1.33)	0.38 (0.21,0.72)	2.72 (1.63,4.54)	0.24 (0.03,1.70)	0.28 (0.07,1.12)	1.97 (0.49,7.90)
Stomach	ref.	1.43 (0.85,2.43)	0.39 (0.18,0.88)	1.13 (0.54,2.37)	1.08 (0.73,1.58)	1.62 (0.84,3.13)	2.12 (1.05,4.26)	1.79 (0.99,3.24)	1.23 (0.17,8.69)
Colorectal	ref.	1.16 (0.87,1.55)	1.13 (0.89,1.43)	0.48 (0.27,0.84)	0.90 (0.73,1.11)	0.80 (0.50,1.27)	0.41 (0.18,0.90)	0.21 (0.09,0.50)	0.97 (0.31,3.02)
Pancreas	ref.	1.03 (0.64,1.67)	0.83 (0.54,1.27)	0.58 (0.26,1.29)	0.73 (0.51,1.04)	1.18 (0.65,2.13)	–	0.32 (0.10,1.00)	–
Lung	ref.	1.55 (1.23,1.95)	1.04 (0.83,1.30)	0.67 (0.43,1.04)	0.59 (0.47,0.75)	0.89 (0.60,1.33)	0.43 (0.21,0.86)	0.03 (0.00,0.23)	0.97 (0.36,2.58)
Breast	ref.	0.67 (0.55,0.83)	0.79 (0.68,0.91)	0.47 (0.34,0.64)	0.66 (0.58,0.75)	1.02 (0.82,1.28)	0.12 (0.06,0.25)	0.37 (0.27,0.51)	0.72 (0.39,1.34)
Uterus	ref.	1.11 (0.64,1.91)	0.93 (0.57,1.50)	0.67 (0.28,1.60)	1.03 (0.72,1.48)	0.60 (0.22,1.60)	–	0.42 (0.14,1.30)	2.17 (0.54,8.73)
Ovary	ref.	0.86 (0.60,1.24)	0.83 (0.62,1.11)	0.28 (0.12,0.62)	0.48 (0.36,0.65)	1.23 (0.82,1.83)	0.60 (0.30,1.21)	0.32 (0.15,0.68)	0.71 (0.18,2.85)
Brain	ref.	0.79 (0.48,1.31)	0.80 (0.53,1.19)	0.74 (0.38,1.42)	1.11 (0.85,1.45)	1.61 (1.00,2.60)	1.39 (0.79,2.46)	0.42 (0.19,0.94)	0.97 (0.24,3.89)
Leukaemia	ref.	0.93 (0.51,1.68)	0.85 (0.52,1.40)	0.66 (0.27,1.58)	0.61 (0.38,0.95)	1.36 (0.70,2.61)	–	0.81 (0.39,1.71)	–
Circulatory	ref.	0.76 (0.64,0.90)	1.20 (1.08,1.34)	0.72 (0.58,0.89)	0.95 (0.86,1.04)	1.19 (1.00,1.42)	1.06 (0.85,1.34)	0.50 (0.38,0.65)	1.47 (0.96,2.26)
Respiratory	ref.	1.08 (0.81,1.44)	0.90 (0.70,1.16)	0.50 (0.30,0.85)	0.70 (0.56,0.87)	1.09 (0.75,1.60)	1.11 (0.70,1.76)	0.65 (0.40,1.05)	1.20 (0.45,3.19)
Digestive	ref.	0.46 (0.30,0.69)	1.70 (1.44,2.02)	0.66 (0.43,1.02)	0.62 (0.50,0.77)	1.20 (0.86,1.69)	0.40 (0.21,0.78)	0.35 (0.20,0.60)	0.54 (0.17,1.68)
Injuries	ref.	0.84 (0.68,1.04)	1.09 (0.94,1.26)	0.57 (0.42,0.79)	0.73 (0.64,0.84)	0.98 (0.75,1.28)	0.47 (0.33,0.68)	0.39 (0.28,0.53)	1.39 (0.93,2.08)
Women – 2000s									
All Deaths	ref.	0.91 (0.85,0.96)	1.16 (1.11,1.22)	0.76 (0.65,0.88)	0.78 (0.74,0.81)	0.94 (0.88,1.01)	0.82 (0.75,0.89)	0.75 (0.70,0.80)	1.18 (1.08,1.29)
Cancer	ref.	0.89 (0.81,0.97)	1.05 (0.98,1.13)	0.78 (0.63,0.97)	0.77 (0.72,0.82)	0.93 (0.84,1.03)	0.62 (0.53,0.71)	0.58 (0.52,0.65)	0.79 (0.67,0.93)
Head and neck	ref.	0.95 (0.52,1.72)	1.75 (1.19,2.57)	2.57 (1.15,5.75)	0.54 (0.32,0.92)	1.10 (0.57,2.12)	0.66 (0.25,1.77)	0.37 (0.14,0.99)	0.54 (0.13,2.16)
Stomach	ref.	0.61 (0.27,1.36)	0.76 (0.41,1.43)	–	2.04 (1.51,2.77)	3.00 (1.94,4.64)	2.32 (1.31,4.10)	1.94 (1.21,3.10)	0.94 (0.30,2.93)
Colorectal	ref.	0.86 (0.62,1.19)	0.97 (0.74,1.27)	1.16 (0.63,2.17)	0.84 (0.67,1.05)	0.80 (0.53,1.19)	0.37 (0.18,0.74)	0.72 (0.50,1.05)	1.22 (0.74,2.00)
Pancreas	ref.	0.65 (0.40,1.06)	1.46 (1.10,1.96)	0.62 (0.20,1.92)	1.12 (0.86,1.45)	1.01 (0.63,1.63)	1.11 (0.64,1.92)	0.90 (0.57,1.42)	0.57 (0.21,1.53)
Lung	ref.	1.12 (0.92,1.35)	1.21 (1.03,1.42)	0.83 (0.51,1.36)	0.66 (0.56,0.78)	0.99 (0.78,1.26)	0.38 (0.24,0.59)	0.19 (0.12,0.30)	0.65 (0.42,1.00)
Breast	ref.	0.75 (0.62,0.90)	0.98 (0.85,1.13)	0.69 (0.45,1.06)	0.65 (0.57,0.74)	0.70 (0.56,0.88)	0.37 (0.26,0.52)	0.69 (0.57,0.84)	0.68 (0.49,0.94)
Uterus	ref.	0.85 (0.47,1.54)	1.60 (1.09,2.34)	1.95 (0.81,4.71)	0.96 (0.65,1.40)	0.90 (0.45,1.80)	1.59 (0.85,2.98)	0.53 (0.24,1.18)	0.27 (0.04,1.88)
Ovary	ref.	1.02 (0.74,1.42)	0.86 (0.63,1.18)	0.99 (0.47,2.09)	0.77 (0.59,0.99)	0.85 (0.55,1.31)	0.78 (0.46,1.32)	0.43 (0.26,0.73)	0.55 (0.24,1.22)
Brain	ref.	0.60 (0.34,1.06)	0.72 (0.46,1.13)	0.97 (0.36,2.59)	0.79 (0.57,1.10)	1.12 (0.70,1.81)	0.88 (0.49,1.60)	0.73 (0.44,1.19)	0.38 (0.12,1.17)
Leukaemia	ref.	1.75 (1.12,2.73)	1.42 (0.93,2.17)	0.86 (0.21,3.44)	0.81 (0.52,1.24)	0.60 (0.25,1.44)	0.91 (0.41,2.03)	0.51 (0.23,1.14)	3.38 (1.99,5.73)
Circulatory	ref.	0.97 (0.84,1.12)	1.08 (0.96,1.22)	0.59 (0.40,0.89)	0.85 (0.77,0.94)	0.90 (0.76,1.07)	1.11 (0.93,1.34)	0.92 (0.79,1.07)	1.24 (0.99,1.55)
Respiratory	ref.	1.03 (0.80,1.31)	0.95 (0.76,1.19)	0.88 (0.48,1.58)	0.69 (0.57,0.85)	0.50 (0.33,0.77)	0.62 (0.39,0.97)	0.72 (0.53,0.98)	1.19 (0.78,1.80)
Digestive	ref.	0.64 (0.47,0.86)	1.51 (1.27,1.79)	0.29 (0.11,0.78)	0.62 (0.51,0.76)	0.98 (0.74,1.30)	0.29 (0.16,0.52)	0.18 (0.10,0.31)	0.88 (0.57,1.35)
Injuries	ref.	0.96 (0.77,1.20)	0.58 (0.45,0.73)	1.01 (0.63,1.62)	0.17 (0.12,0.24)	0.81 (0.62,1.06)	0.80 (0.61,1.06)	0.83 (0.67,1.02)	0.86 (0.62,1.21)

All models are adjusted for attained age

When zooming in on the broad categories of COD, we observed that cancer mortality was the most common COD for both native Belgians and migrant groups. Compared with the migrant groups, cancer mortality was high among native Belgian men and women. Cancer mortality rates were lower among men than among women, and were lowest among migrants from Turkish and Moroccan descent (Tables 3 and 4). For instance in the 2000s, Turkish, Moroccan and SSA men had in relative terms respectively 36% (MRR: 0.64; 95% CI 0.56–0.72), 39% (MRR: 0.61; 95% CI 0.56–0.66) and 29% (MRR: 0.71; 95% CI 0.61–0.84) lower cancer mortality compared with native Belgians (Table 5). In women similar migrant mortality advantages were observed for cancer mortality. The second most common category of death among men and women were circulatory diseases. Yet, the proportion of overall mortality due to these circulatory diseases was larger in the 2000s among native Belgians compared with some migrant groups, e.g. French and Spanish men and women (Tables 3 and 4). In relative terms, most migrant groups had lower mortality from circulatory diseases compared with native Belgians, except during the 1990s when French women and Eastern European men and women had mortality excesses of respectively 20% (MRR: 1.20; 95% CI 1.08–1.34), 16% (MRR: 1.16; 95% CI 1.04–1.30) and 19% (MRR: 1.19; 95% CI 1.00–1.42), which disappeared in the 2000s (Tables 5 and 6). Likewise, mortality from respiratory diseases, digestive diseases and injuries was generally higher among native Belgians, with only some exceptions. For instance, men and women of French descent had both in the 1990s as in the 2000s elevated mortality from digestive diseases with MRRs in the 2000s of respectively 1.28 (95% CI 1.12–1.46) and 1.51 (95% CI 1.27–1.79) (Tables 5 and 6).

### Differences in cancer-specific mortality by migrant origin

Because of our special interest in cancer, we also studied the most common subsites of cancer. The most common causes of cancer deaths were lung cancer for men and breast cancer for women, and this pattern was similar in all migrant groups. For most cancer sites, native Belgians had higher mortality rates compared with the migrant groups. Again, the main exception were migrant men and women from French descent. In the 2000s, French migrants in particular had higher mortality from alcohol-related cancers such as cancers of the head and neck (MRR_men_ 1.67; 95% CI 1.40–1.99 and MRR_women_ 1.75; 95% CI 1.19–2.57) and liver (MRR_men_ 2.21; 95% CI 1.69–2.88) (Tables 5 and 6. In contrast to the general pattern, native Belgians had advantageous stomach cancer mortality rates compared with some migrant groups, especially among women. For instance, in the 2000s, women from Italian, Turkish and Moroccan descent had twice as much risk, and women of Eastern European descent even threefold as much risk to die from stomach cancer compared with native Belgian women (Table 6). In the 2000s, besides French migrant men, both Italian and SSA men also had higher mortality from liver cancer, with MRRs of respectively 1.51 (95% CI 1.21–1.89) and 4.16 (2.78–6.23) (Table 5). Belgian men and women had particular high lung cancer mortality rates. In the 2000s, the only migrant groups showing higher lung cancer mortality were Eastern European men (MRR: 1.24; 95% CI 1.08–1.42) and French women (MRR: 1.21 (95% CI: 1.03–1.42) (Tables 5 and 6). In women, especially Turkish and Moroccan women had a clear mortality advantage with respectively 62% (MRR: 0.38; 95% CI: 0.24–0.59) and 81% (MRR: 0.19; 95% CI: 0.12–0.30) lower lung cancer mortality in the 2000s compared with native Belgian women (Table 6). In the 2000s, SSA African men had an elevated prostate cancer mortality rate compared with native Belgians, while SSA women had elevated leukaemia cancer mortality rates. For the most common cause of female cancer death, breast cancer, we observed a clear mortality advantage in both periods among women with a migrant origin. In the 2000s, Turkish women even had 63% lower breast cancer mortality compared with native Belgian women (MRR 0.37 (0.26–0.52)) (Table 6). Finally, women from French descent had 60% higher mortality from uterus cancer compared with native Belgian women (MRR: 1.60; 95% CI 1.09–2.34) (Table 6).

### Evolution between the 1990s and 2000s of (differences in) mortality by migrant origin

Among Belgian men as well as all Western European men, overall mortality declined over time, yet among Turkish and SSA men the trend was rather stable (Table 3). For Belgian and Western European men, mortality decreased for injuries, circulatory diseases, respiratory diseases and cancer, yet remained stable for digestive diseases. In Belgian women, an overall mortality decline was observed, yet this trend was stable among women from French, Spanish and Turkish descent (Table 4). In Belgian women, mortality due to injuries, circulatory diseases and cancer decreased as in men, while mortality from respiratory diseases increased and mortality from digestive diseases decreased, unlike men (Table 4). Cancer mortality decreased also among migrant women from Dutch and Eastern European descent while it remained stable over time among the other migrant groups. Furthermore, among most Western-European women, mortality from circulatory diseases and injuries decreased. In contrast, among Moroccan women, mortality from circulatory diseases increased between the 1990s and the 2000s.

The evolution of cancer mortality in Belgian men was favourable for most cancer sites, with the exception of liver cancer which increased over time (Table 3). In women, the site-specific cancer mortality trends were also favourable except for lung cancer mortality which increased with 38% (Table 4). In contrast to their high mortality levels, head and neck cancer mortality decreased over time with 45% for men of French descent (Table 3). As observed in Belgian women, lung cancer mortality increased with 59% among French migrant women (Table 4).

## Discussion

### Strengths and weaknesses

Belgium is a country with a high proportion of migrants [3–5], and therefore particularly apt to study mortality differences by migrant origin. The results presented in this paper are based upon an exhaustive, nationwide dataset consisting of an individual linkage between Census and Register data. As a result of this individual linkage, a numerator-denominator bias was avoided. This allowed us to precisely assess the evolution over time in mortality patterns for all major COD, breaking down by gender and major groups of origin. We were able to assess the evolution over time by comparing the migrant mortality differentials in the 1990s with the 2000s. Yet, due to the administrative nature of the dataset, we did not obtain information on the different exposures migrants had faced throughout their life course [7, 13]. The dataset consists of all Belgian residents at the time of the census, without allowing new immigrants to join the dataset. People are followed-up until emigration, death or end of follow-up. Yet, we cannot fully exclude a salmon bias in the case of unreported emigration [11]. A study in Sweden estimated that 10% of the immigrants that return to their home country do not report this to keep their option open to come back if necessary, for instance when they want to make use of the health system [9], although this remigration occurs mainly among migrants aged 65+ [20], which is not the studied population. Moreover, Vandenheede and colleagues proved that it is unlikely that the observed migrant mortality patterns in Belgium are explained by unregistered emigration of ill immigrants [14]. We decided to stratify our analyses by gender and migrant origin as these groups may have different reasons to migrate though this information was not available in the dataset. The analyses are performed for all important COD, in order to gain as much knowledge on the various mechanisms at play. However, we did not dispose of additional information on morbidity, nor on lifestyle, use of health care and so on. Adding such information to the analyses could undoubtedly deepen our knowledge on the mechanisms at play. Even though we included the total Belgian population within the age range of 25–65 years, as a result of the stratification by gender and migrant origin, for some COD the numbers of deaths are quite small. Because of this, we decided not to distinguish between first and second-generation migrants. Doing so might provide additional useful information, but especially in the 1990s, the number of deaths among second-generation migrants was too low because of their young ages. Similarly, we decided not to adjust for additional migration variables such as length of stay or age at migration. We performed single-comparison analyses, showing differences in mortality between the migrant and native population. Yet, performing a two-comparison method by comparing mortality of the natives in the home country, the natives in the host country and the immigrants would be interesting as well [24], yet not feasible with our dataset.

### Reflections on the main findings of the study

We generally observed a migrant mortality advantage for overall, cause-specific and cancer-specific mortality. This finding is in line with literature on mortality differentials in migrants [1, 3, 5, 6, 9, 11, 16, 17, 22, 25, 26]. This migrant mortality advantage may be explained by a combination of factors. The first hint at the mechanisms behind is the mortality pattern itself. Although there was a general mortality advantage for migrants, we observed some variation by COD. For cancers of the stomach and the liver for instance, the pattern was reversed showing excessive mortality among most migrant groups, as previous studies also noted [1, 3, 6, 18, 22, 26]. These cancers are infection-related, which occur more often among non-western populations [6, 16, 26]. An established risk factor for stomach cancer is infection with Helicobacter pylori, due to unfavourable hygienic and living conditions in childhood [6]. However, part of the stomach cancers, i.e. cancers of the cardia, are related to lifestyle. Unfortunately, we were not able to distinguish cardia from non-cardia stomach cancers due to small numbers and the high proportion of unspecified stomach cancers. On the other hand, liver cancers are associated with viral infections such as hepatitis B and C during early childhood [16, 22], which is likely to explain the excess liver cancer mortality among SSA men [10]. Nonetheless, mortality in western societies and Belgium is mainly driven by lifestyle-related COD such as cancers (lung cancer in men and breast cancer in women), and cardiovascular mortality, which might explain the relative small impact of the excess infection-related mortality among migrants on the picture as a whole [18]. For the most common COD, migrants (especially non-western) had a mortality advantage. For lung cancer for example, we clearly observed that native Belgians, French men and women, as well as Dutch women died more often compared with the other migrant groups.

The second explanatory factor at play is the fact that migrants, especially non-western migrants, have a healthier lifestyle compared with native Belgians and western populations and hence a lower risk for lifestyle-related COD and cancers [1, 3, 6, 11, 16, 17, 25]. The western lifestyle is characterized by high levels of physical inactivity and a poor diet with low vegetable and fruit intake which is related to e.g. cardiovascular mortality or colorectal cancer; by tobacco and alcohol consumption which is associated with cancers of the lung and head and neck; and by the postponement of reproductive behaviour which is related to breast cancer mortality. The migrant mortality advantage was particular strong for the non-western groups of Turkish and Moroccan migrants, as was observed in previous research [5]. Turkish and Moroccan women tend to be younger at first pregnancy and to have more children, which are protective factors against breast cancer [17, 26]. Moreover, Turkish and Moroccan men and women tend to have lower levels of alcohol consumption due to their religious beliefs [17, 27], and may keep up their Mediterranean diet with a high fruit and vegetable content, at least shortly after migration [10, 11]. These healthy practices may operate as a protective factor against the detrimental effects from other health-damaging practices [11]. For instance, low alcohol consumption may buffer the negative effects of smoking on lung cancer. Another explanation for the lower lung cancer mortality may be that notwithstanding a high proportion of smokers, they may have a lower amount consumed per person [18]. In contrast, migrants from French and Eastern European descent were exceptions to this general pattern and generally showed higher mortality rates compared with native Belgians. Migrants from French descent consistently had higher mortality, particularly among men. This is in line with previous findings showing high smoking- and alcohol-related mortality among men living in northern France [28]. Given the fact that French migrants generally live in the border regions, some cross-border overlap of lifestyle is very likely [2, 14]. Migrants from Eastern Europe also experienced excess mortality, mainly due to lung cancer mortality which can be explained by the high levels of smoking among Eastern Europeans [29].

This brings us to the third explanation given in literature, i.e. the mortality advantage is the result of a health selection effect: i.e. the fittest and healthiest individuals immigrate [3, 11, 22]. This health selection into migration is especially applicable in the case of labour migration. Hence, if there would be a health selection effect we could expect that the effect wears off with time and that it may not be as strong for women than for men [10, 11, 17, 22]. Men and women have a different migration trajectory: men who used to migrate for work purposes had to be in good health and are therefore more likely to have received medical health checks at the workplace. Women on the other hand used to immigrate for family reunification reasons and often did not work outside the home. This could explain the fact that the mortality differences between migrants and native Belgians are generally smaller for women than for men. This selection may even be reinforced by return migration of sick immigrants to their home country [11]. However, this might be in contrast with the fact that migrants most often have settled with their families, and that, especially for non-western migrants, the health care system may be better organized in the host country [1, 21, 26]. Yet, this does not exclude the fact that migrants may experience barriers in terms of access to health services and treatment, e.g. in terms of language, finances, risk perception or knowledge [7, 21, 30, 31]. For instance, the higher prostate cancer mortality among SSA men may point to inequalities in health care access as survival from this cancer is associated with early diagnosis [32]. The healthy migrant effect should also dilute over time because of the adaptation of western lifestyle as they longer reside in the host country [10, 13, 15, 17]. Yet, both in the 1990s and the 2000s, clear mortality advantages could be observed, which suggest that selection is unlikely to be the main explanatory factor [1, 3].

A final factor to explain the migrant mortality advantage is the different genetic makeup of the various migrant groups [3, 17, 33]. For instance, genes are involved in part of the breast cancer cases and may therefore be part of the observed breast cancer differences [3]. Furthermore, the elevated prostate cancer mortality among SSA men may also be partly due to genetic factors [16]. Previous research [17] also suggested that lower lung cancer mortality patterns among Moroccan migrants may also be explained by protective genetic characteristics.

Overall mortality declined over time among native Belgian and western-European migrant men, which was mainly due to declines in injuries and circulatory diseases. Among women, overall mortality declined among native Belgians, yet remained stable over time among French, Spanish and Turkish migrant women. Among French women, this trend was partly due to the rise in lung cancer mortality, which was also apparent among native Belgian and Italian women. In contrast, among Belgian and western European men, lung cancer mortality decreased while among non-western European men no decrease was noted between the 1990s and the 2000s. Turkish women on the other hand, experienced a rise in breast cancer mortality. These evolutions over time are likely to be the result of the adaptation of the western lifestyle by non-western immigrants, i.e. smoking and the postponement of childbearing [3, 26].

## Conclusions

The finding that most migrant groups have lower mortality compared with the native Belgian population proves there is room for improvement in the field of public health in Belgium [3, 14]. The mortality advantage is currently highest for lifestyle-related diseases. Therefore, policy-makers should focus on primary prevention measures for native Belgians in order to ameliorate the health behaviours within this group. At the same time, we must bear in mind that even though the migrant mortality advantage still persists, it may diminish or even disappear in the future at least for some migrant groups considering the lag time for instance between smoking and mortality from some cancer sites [13, 26, 32]. This suggests that preventative efforts should be continuously taken to discourage smoking and to encourage to preserve the healthy lifestyle of the home country, especially among the most vulnerable groups in terms of unhealthy behaviours [22, 34]. To identify these groups, future studies should go into more depth and, if possible, disentangle the migrant groups by important characteristics such as migrant generation, duration of residence in the host country and SEP [13]. Besides preventative measures, efforts should be made to ensure access to health care among the social and cultural strata [6, 7, 21, 30, 34, 35].

Important questions remain unanswered and should be further studied. For instance, how much of the observed mortality differences can be attributed to the SEP of the migrants? How do the mortality patterns of the immigrants relate to the mortality patterns in their home country? To what extent are morbidity patterns similar to the observed mortality patterns? Previous research observed a morbidity-mortality paradox [1, 36]. As mortality is an indicator of the fatality of diseases and of access to care, mortality patterns are not necessarily a reflection of the health patterns in society [3]. Therefore, future studies should probe into morbidity differences between native and migrant populations as well, ideally using a life course approach [13, 22]. Doing this will provide information on the importance of different exposures at certain times and on the clues about the genetic, socioeconomic, cultural or environmental nature of these differences [13]. Therefore, in future studies, we will use record-linkage data on cancer incidence and survival by migrant origin to probe into the origins of the observed differences in cancer mortality.

## Abbreviations

ASMR: Age-standardized mortality rate
COD: Causes of death
MRR: Mortality rate ratio
SEP: Socioeconomic position
SSA: Sub-Saharan Africa
